# Review of 16S and ITS Direct Sequencing Results for Clinical Specimens Submitted to a Reference Laboratory

**DOI:** 10.1155/2016/4210129

**Published:** 2016-03-01

**Authors:** Michael Payne, Robert Azana, Linda M. N. Hoang

**Affiliations:** ^1^Department of Pathology and Laboratory Medicine, UBC, Vancouver, BC, Canada V6T 2B5; ^2^BC Centre for Disease Control Public Health Laboratory, Vancouver, BC, Canada V5Z 4R4

## Abstract

We evaluated the performance of 16S and internal transcribed spacer (ITS) region amplification and sequencing of rDNA from clinical specimens, for the respective detection and identification of bacterial and fungal pathogens. Direct rDNA amplification of 16S and ITS targets from clinical samples was performed over a 4-year period and reviewed. All specimens were from sterile sites and submitted to a reference laboratory for evaluation. Results of 16S and ITS were compared to histopathology, Gram and/or calcofluor stain microscopy results. A total of 277 16S tests were performed, with 64 (23%) positive for the presence of bacterial DNA. Identification of an organism was more likely in microscopy positive 16S samples 14/21 (67%), compared to 35/175 (20%) of microscopy negative samples. A total of 110 ITS tests were performed, with 14 (13%) positive. The yield of microscopy positive ITS samples, 9/44 (21%), was higher than microscopy negative samples 3/50 (6%). Given these findings, 16S and ITS are valuable options for culture negative specimens from sterile sites, particularly in the setting of positive microscopy findings. Where microscopy results are negative, the limited sensitivity of 16S and ITS in detecting and identifying an infectious agent needs to be considered.

## 1. Introduction

Rapid identification of pathogens from clinical specimens is important for the selection of correct treatment, as well as for patient prognosis. However, in some specimens cultures remain negative and are ideal candidates for use of molecular methods for amplification and identification of the potential pathogens [1]. The 16S rRNA gene in bacteria and the 18S rRNA gene, with associated internal transcribed spacer (ITS) regions, in fungi are common gene targets used in the microbiology laboratory for gene amplification followed by sequencing for organism identification. These assays were initially developed as a method for the identification and classification of organisms from culture specimens [2–4]. However, these assays have been shown to successfully amplify and identify pathogens from clinical specimens where cultures are negative, and organisms may be fastidious or nonviable from antibiotic exposure [1].

Amplification of 16S and ITS DNA directly from clinical specimens has both challenges and limitations. Polymicrobial infections or nonsterile sites typically cause difficulties interpreting sequencing results [5]. Specificity of results may also be difficult to determine as contamination of the sample can occur during collection or in the laboratory. Also, amplification of microorganism DNA from the sample may not necessarily ensure the organism identified is the causal pathogen. The negative predictive value of 16S and ITS is difficult to determine and may not be ideal, particularly in smear negative samples [1]. Also, 16S and ITS are labour-intensive procedures requiring advanced infrastructure and technical expertise and can add considerably to the workload of a laboratory [5]. Finally, standardization for 16S and ITS is poor, with many different primer/probe targets, specimen processing methods, and reference databases in use [6, 7].

Given the lack of information on test performance from direct clinical specimens, the results of 16S and ITS tests performed over the past 4 years were reviewed. Through this review we aimed to better define the correlation with microscopy results and overall performance of 16S and ITS testing from direct clinical specimens.

## 2. Materials and Methods

Since 2008, the British Columbia Centre for Disease Control Public Health Laboratory (PHL) has offered 16S and ITS PCR and sequencing directly from clinical specimens. These tests are offered to enhance pathogen detection from patient specimens in acute care facilities in British Columbia, on a referral basis. A retrospective review of testing results from January 2009 until June 2013 was conducted. Results from patients were stored in a secure database, along with microscopy and culture results. All tests performed during this time period were included in the study.

Prior to testing, consultation with the program medical microbiologist at the PHL was required. The key parameters for test approval included negative culture for pathogen, specimen from sterile site, high index of suspicion for infection from clinical presentation, and other diagnostic testing. Priority was given to specimens with positive microscopy and patients previously treated with antibiotics. Fresh tissue/fluid specimens were preferred, but formalin fixed paraffin embedded (FFPE) samples were accepted, if no fresh specimen was available. Specimens were submitted from referring hospitals using routine specimen transfer methods. Samples from FFPE tissue were sent by their respective pathology departments, typically with 5–7 scrolls of tissue. Samples for 16S PCR/sequencing underwent DNA extraction using the QIAamp DNA Mini Kit (Qiagen, Valencia, CA). Product manual protocols were followed for extraction of tissues, body fluids, and blood (https://www.qiagen.com/us/resources/resourcedetail?id=67893a91-946f-49b5-8033-394fa5d752ea&lang=en). However, DNA was eluted into 100 *μ*L, instead of 200 *μ*L, in order to improve DNA concentration. FFPE specimens underwent an additional extraction procedure with PRO-PAR solution (http://www.anatechltdusa.com/productlit/proparclearant.html) prior to routine PCR extraction. For ITS sample extraction, the UltraClean® Microbial DNA Isolation Kit was used (MO BIO Laboratories, Carlsbad, CA).

16S rDNA PCR amplification was performed using the 2 primers: 8-27F 5′-AGA GTT TGA TCA TGG CTC AG-3′ and 519-536R 5′-GWA TTA CCG CGG CKG CTG-3′, which generates 500 bp amplicon [8, 9]. ITS rDNA PCR amplification was performed using the 2 primers: ITS1 5′-TCC GTA GGT GAA CCT GCG G-3′ and ITS4 5′-TCC TCC GCT TAT TGA TAT GC-3′ [10]. The fragment length of ITS amplification was variable, 550–800 bp. Following amplification of the 16S and ITS regions, the samples were run on a 2% agarose gel to identify those with amplified product. Known samples of* Aspergillus flavus* and* Ornithobacterium rhinotracheale* were used as positive controls for ITS and 16S, respectively. Only those specimens with amplified product, of the appropriate size, were selected for sequencing. PCR products were then purified with the Qiagen QIAquick PCR Purification Kit (Qiagen, Valencia, CA). They were again amplified using forward and reverse primers for the 16S and ITS regions with the BigDye® Terminator v3.1 Ready Reaction Mix*™* (Applied Biosystems, Foster City, CA, USA). Products from this second amplification were then purified with the BigDye® XTerminator*™* Purification Kit (Applied Biosystems) to remove unincorporated dye terminators. Samples were then sequenced using the ABI Prism 3130xl Genetic Analyzer (Applied Biosystems). Sequence files were then evaluated using the ABI Sequencing Analysis and Sequence Scanner Software. Sequences were then aligned using Geneious Pro*™* (Biomatters, Auckland, New Zealand).

Assembled 16S sequences underwent the BLAST procedure, for RefSeq (http://www.ncbi.nlm.nih.gov/refseq/), 16S RDP (http://rdp.cme.msu.edu/) and Euzéby taxonomy list (http://www.bacterio.net/-bmsab.html). Interpretations were guided by the CLSI document MM18A [11]. However, all results were reviewed by the program's medical microbiologist and the decision on final identification was based on correlated clinical judgement. Assembled ITS sequences were loaded into the NCBI GenBank database and underwent the BLAST procedure (http://blast.ncbi.nlm.nih.gov/Blast.cgi). These were then interpreted using maximum bit score, percent coverage, and *E*-value. Since there are no curated ITS sequences, GenBank database sequences were used, but preference was given to type strain entries. Again, final interpretations were based on criteria from the CLSI document MM18A [11]. Most samples had cultures and microscopy (Gram stain, calcofluor stain, and histopathology) performed at the referring laboratory sites, according to their standard protocols. Reports were accessed using a common laboratory information system, if available. Some specimens did not have microscopy or culture results available, but these specimens were still included in the analysis.

Ethical review and approval was obtained from the University of British Columbia as well as the other health authorities involved in the study.

## 3. Results

During the 4-year study period, a total of 277 clinical specimens underwent 16S rDNA testing. These specimens were from 216 patients. The number of 16S specimens received in 2009 was 43, 47 in 2010, 55 in 2011, 86 in 2012, and 46 in the first 6 months of 2013. Specimens were composed of 148 fresh tissues, 122 sterile fluids, and 7 paraffin embedded samples. Of the fresh tissue samples there were 24 bone, 22 cardiac, 18 synovial, 17 CNS, 11 abdominal, 10 lymph node, 6 lung, and 40 miscellaneous tissues. The most common sterile fluids included 62 CSF, 26 joint fluids, 12 pleural fluids, 10 direct blood cultures, and 12 miscellaneous fluids.

Overall, 64 (23%) of specimens from 58 (27%) patients were positive for amplified product (Table 1). Identification of an isolate was more likely in microscopy positive 16S samples 14/21 (67%), compared to 35/175 (20%) of microscopy negative samples. Additionally, if the Gram smear showed signs of inflammation, determined by presence of white blood cells, the yield was increased from 5/45 (11%) specimens versus 27/110 (25%) specimens. However, the presence or absence of inflammation was not available for all specimens. The morphologic microscopy results correlated with all 14 of the specimens positive for both microscopy and 16S.

Of the fresh tissue samples, 5/24 (21%) bone, 3/22 (14%) cardiac, 2/18 (11%) synovial, and 4/17 (24%) CNS tissues were positive for amplified product. For sterile fluids, 12/62 (19%) CSF, 4/26 (15%) joint fluids, 6/12 (50%) pleural fluids, and 2/10 (20%) direct blood cultures had an identified amplified product.

There were 62 specimens positive for a unique identified amplified product, with repeat positive specimens removed. Streptococci were by far the most common, with 17/62 (27%). The species isolated included 5* S. pyogenes*, 4 viridans group streptococci, 2* S. agalactiae*, and 2* S. pneumoniae*. Of note, in 4 cases 16S was unable to resolve* S. pneumoniae*/*S. mitis*/*S. pseudopneumoniae* to species level. A large proportion of positive isolates included atypical bacteria 8 (13%). In addition there were also 8 (13%) specimens positive for anaerobic bacteria.

A total of 110 specimens underwent ITS rDNA testing, from 64 patients. The number of ITS specimens received in 2010 was 20, 24 in 2011, 44 in 2012, and 22 in the first 6 months of 2013. Of the 110 specimens tested, there were 14 (13%) positive. An additional 7 (6%) specimens were positive for rare environmental fungal species and reported as such.

Specimens were composed of 43 fresh tissues, 34 sterile fluids, and 33 FFPE tissues (Table 2). Fresh tissue specimens had amplified product in 6/43 (14%), and sterile fluids yielded 3/34 (9%). Of the 33 FFPE tissues 5 had amplified product (15%). The yield of microscopy positive ITS samples, 9/44 (21%), was higher than microscopy negative samples 3/50 (6%). The morphologic microscopy results correlated with all 9 samples positive for both microscopy and ITS.

Of the 14 specimens positive for identified potential pathogens, 3 were repeat positives. The 11 remaining unique positive ITS samples identified a broad range of fungal species. There were five patients with yeast identified:* Candida albicans* (1),* Candida lipolytica* (2),* Rhodotorula mucilaginosa* (1), and* Cryptococcus neoformans* var.* grubii* (1). There were four patients with moulds identified:* Aspergillus nidulans* (1),* Rhizopus* spp. (2), and* Massarina* spp. (1). The dimorphic mould,* Histoplasma capsulatum*, was identified in 2 separate patients.

## 4. Discussion

This study has reviewed 16S and ITS testing from direct clinical specimens for a 4-year period at the PHL. We found that the number of tests performed has increased yearly, for both 16S and ITS, since they were introduced in 2009 and 2010, respectively. This likely reflects increasing knowledge and value of the test by clinicians, as well as increasing overall patient volumes. The testing volumes for 16S are double those for ITS, which may be due to the smaller patient population at increased risk of fungal infections as well as the lower incidence of invasive fungal versus bacterial infections.

Overall, 16S testing of culture negative specimens at our centre had a positivity rate of 23%; this is comparable to previous studies, reviewing a wide variety of clinical samples [12–14]. One important factor predicting success was a positive microscopy result, either by Gram smear or by histology. This has been noted as a positive predictive factor previously [1, 15]. There were 35/175 specimens which were 16S positive but were negative on microscopy for a pathogen. Of these, 152 specimens had information on inflammation from the microscopy report (presence of white blood cells). For the 16S positive samples, 25/29 (86%) had microscopy results positive for inflammation. For the specimens with negative 16S results, 83/123 (67%) had signs of inflammation. Therefore, the specimens positive for 16S were more likely to have signs of inflammation, compared to 16S negative samples, in the setting of microscopy negative for bacteria. Also of note, 4/35 16S positive, microscopy negative samples were positive for a pathogen, which would not stain on routine Gram smear.

Specimens for 16S were composed mostly of fresh tissues and sterile fluids. Of the fresh tissues, bone and joint samples were the most common, followed by cardiac and CNS tissues. Of the sterile fluids, over half were CSF, followed by joints fluids. This is a similar distribution of specimens, compared with previous reviews [5, 12].

There have been a large number of studies focusing on the use of 16S for particular specimen types. For bacterial meningitis, a recent meta-analysis of 14 studies has shown a 30% positivity rate of 16S in culture negative presumed bacterial meningitis [16]. In this review, our positivity rate was comparable at 20% for CSF samples and was not improved with microscopy positive CSF samples. Bone and joint tissue/fluid specimens are one of the most common specimens submitted for 16S and have been previously studied [17–20]. In these studies, 16S has performed well, compared to cultures, with the added benefit of identifying atypical species. Jensen et al. [12] found a positivity rate of 16% for bone and joint specimens, similar to the 15% and 17% positivity rate for synovial fluids and bone/joint specimens, respectively, in this study.

Streptococci were the most common group of bacteria recovered. Previous studies, reviewing 16S for a variety of specimens types, also found that streptococci were the most common species recovered [12–14]. This may be due to the fact that streptococci are typically more susceptible to antibiotics, as a group, and are therefore more likely to be culture negative after initial treatment with antibiotics. Alternatively, this may just reflect the fact that streptococci are one of the more common isolates in a microbiology laboratory. Of note, there were a large number of specimens found to include atypical and anaerobic species. These groups are typically more difficult to culture and may explain their high prevalence in culture negative specimens referred to our laboratory. Without 16S the etiologic agent may have went undiagnosed in these cases.

In contrast to 16S specimens, ITS had a much larger proportion of FFPE tissues. The reasons for this are unclear but may be due to increased ease of visualization of fungal hyphae during histologic examination, compared to bacteria, which would prompt a pathologist to request ITS testing. Of the 30 ITS FFPE specimens with histology results, 20 (67%) had histology positive for fungal elements. Due to the small number of specimens for ITS testing, subgroup analysis of clinical specimen types was not performed.

All ITS specimen types (FFPE, fresh tissues, and sterile fluids) had lower recovery rates, compared to 16S. This could be a reflection of a lower rate of invasive fungal disease, compared to bacterial infections. Alternatively, ITS may not be as sensitive as 16S for detecting disease; this is possible as ITS is a newer, less standardized technology [7]. Recovery was improved with ITS specimens that were microscopy positive compared to negative samples. Most of this difference was due to the increased yield with microscopy positive fresh tissue samples, which was not seen in the FFPE tissues. The positivity rate in our review is lower than other studies reported previously [21–23]. They found a positivity rate of 89–100% and 64%–80% compared to culture positive and histology only positive samples, respectively. The lower overall positivity rates found in our study may be a result of different patient populations. Also, our study tested sterile fluids, which had a lower positivity rate compared to tissues. Despite its low yield in our study, ITS is still a useful technology as identification to genus or species level purely by histologic appearance can be unreliable in culture negative samples [24].

Our study seeks to better understand the test performance of 16S and ITS at a reference laboratory. Its strengths include a large number of both 16S and ITS samples over a 4-year span from a diverse patient population, correlation to microscopy results, and stratification by specimen type. Our study has several limitations. There was a lack of clinical information for patients, particularly information on pretreatment with antibiotics and patient outcomes. Without clinical information, it is difficult to interpret the significance of specimens with negative microscopy and positive for bacterial/fungal DNA. We also did not have access to microscopy results for all specimens, due to separate laboratory information systems. In addition, patients were selected for testing based on high likelihood of infection; therefore our results would not reflect the test performance in the general patient population. In general, the performance of 16S is not uniform for all bacterial groups, with some requiring secondary target PCR for speciation. However, in the samples tested at the PHL, 16S was sufficient for bacterial identification.

In conclusion, molecular methods are increasingly being used for the diagnosis of infections, which is reflected in the increased testing volumes in our study. Direct specimen testing with bacterial 16S and fungal ITS has important roles in the microbiology laboratory, particularly in patients with previous antibiotic treatment, microscopy positive/culture negative samples, and if there is a suspicion of atypical/fastidious pathogens. However, our low recovery rate, particularly in ITS samples, highlights the need for careful selection of appropriate samples and interpretation of results. Based on our findings, 16S and ITS should not be used as tests to rule out infection; however, these assays are helpful if positive.

## Figures and Tables

**Table 1:** Amplification and identification rates for direct 16S testing.

Specimen type	Number of positive specimens	Number of negative specimens	Percent positive	Total number of specimens
Fresh tissue				
Microscopy positive^a^	9	5	64.3%	14
Microscopy negative	18	88	17.0%	106
No microscopy result	6	22	21.4%	28
Total	33	115	22.3%	148
FFPE^b^ tissue				
Microscopy positive	3	0	100.0%	3
Microscopy negative	0	2	0.0%	2
No microscopy result	0	2	0.0%	2
Total	3	4	42.9%	7
Sterile fluids				
Microscopy positive	2	2	50.0%	4
Microscopy negative	17	50	25.4%	67
No microscopy result	9	42	17.6%	51
Total	28	94	23.0%	122
All specimens types				
Microscopy positive	14	7	66.7%	21
Microscopy negative	35	140	20.0%	175
No microscopy result	15	66	19.8%	81
Total	64	213	23.1%	277

^a^Note: not all specimens had microscopy results available.

^b^Formalin fixed paraffin embedded.

**Table 2:** Amplification and identification rates for direct ITS testing.

Specimen type	Number of positive specimens	Number of negative specimens	Percent positive	Total number of specimens
Fresh tissue				
Microscopy positive	6	13	31.6%	19
Microscopy negative	0	20	0.0%	20
No microscopy result	0	4	0.0%	4
Total	6	37	14.0%	43
FFPE^a^ tissue				
Microscopy positive	2	18	10.0%	20
Microscopy negative	2	8	20.0%	10
No microscopy result	1	2	33.3%	3
Total	5	28	15.2%	33
Sterile fluids				
Microscopy positive	1	4	20.0%	5
Microscopy negative	1	19	5.0%	20
No microscopy result	1	8	11.1%	9
Total	3	31	8.8%	34
Total for all specimens				
Microscopy positive	9	35	20.5%	44
Microscopy negative	3	47	6.0%	50
No microscopy result	2	14	12.5%	16
Total	14	96	12.7%	110

^a^Formalin fixed paraffin embedded.
